# At the heart of change: Differences in young offenders’ HRV patterns after the delivery of the PSYCHOPATHY.COMP program

**DOI:** 10.3389/fpsyt.2022.1032011

**Published:** 2023-01-10

**Authors:** Rúben Sousa, Diana Ribeiro da Silva, Nicola Petrocchi, Paul Gilbert, Daniel Rijo

**Affiliations:** ^1^Center for Research in Neuropsychology and Cognitive Behavioral Intervention, Faculty of Psychology and Educational Sciences, University of Coimbra, Coimbra, Portugal; ^2^Department of Economics and Social Sciences, John Cabot University, Rome, Italy; ^3^School of Allied Health and Social Care, College of Health and Social Care, University of Derby, Derby, United Kingdom

**Keywords:** PSYCHOPATHY.COMP, compassion focused therapy (CFT), clinical trial, conduct disorder (CD), emotion regulation, heart rate variability, male young offenders

## Abstract

**Introduction:**

Literature has pointed the need for intervention programs specifically tailored to target the treatment needs of young offenders, as well as the need to test the efficacy of such programs through physiological indexes of emotion regulation (e.g., heart rate variability; HRV), complementing self-reports typically used as outcome measures. The PSYCHOPATHY.COMP is a 20-session individual intervention program based on Compassion Focused Therapy aiming to reduce psychopathic traits and disruptive behavior among young offenders through the development of a compassionate motivation, while stimulating the soothing system as a strategy to improve emotion regulation. Previous research with young offenders has shown decreases in vagally mediated HRV (vmHRV) when the soothing system is activated. This physiological pattern seems to mirror threat-like responses that contrast with relaxed states.

**Methods:**

To test the efficacy of the PSYCHOPATHY.COMP, a clinical trial was implemented encompassing a treatment (*n* = 56) and a control group (*n* = 53). Treatment participants attended the PSYCHOPATHY.COMP, while controls received the Treatment As Usual (TAU) delivered in Portuguese juvenile detention facilities. HRV data was collected throughout a standardized procedure (encompassing resting, reactivity and recovery phases) specifically designed to trigger the soothing system. Participants were assessed at pre-treatment, post-treatment and 6-months follow-up.

**Results:**

Although treatment participants continued to process the soothing system as unpleasant (with decreased vmHRV), they seem to become able to adaptively recover from the stimuli without avoiding it or resorting to maladaptive coping strategies. The physiological pattern was in line with participants’ decreases in difficulties in emotion regulation across the assessment periods. In contrast, controls seemed to have actively employed coping strategies associated with increases in vmHRV not only when the soothing system was triggered, but also when recovering from the stimuli. Congruently, for controls, increases in difficulties in emotion regulation were found, with increases in the lack of emotional clarity across the assessment periods.

**Discussion:**

Findings offer new evidence for the efficacy of the PSYCHOPATHY.COMP program in improving emotion regulation in young offenders, assessed through both self-report and physiological measures. Additionally, findings support the assessment of the autonomic balance as a treatment efficacy index in future research, targeting the rehabilitation of these youth.

**Clinical trial registration:**

ClinicalTrials.gov, identifier NCT03971682.

## 1. Introduction

Detained youth with conduct disorder (CD) are considered an at-risk and difficult to treat population, especially if they present high levels of psychopathic traits (1–3). Although there is an urgent need to offer them specialized interventions, research on the topic is still scarce and methodologically flawed [see (4–7)]. However, the recently developed PSYCHOPATHY.COMP program (for a detailed description of the program, see Section “Interventions”) is the first individual program specifically designed to reduce antisocial behavior and psychopathic traits through the promotion of a compassionate motivation in young offenders (8, 9). This program has showed to be effective in reducing psychopathic traits and disruptive behavior, while promoting a compassionate motivation among male detained youth (10–12). Despite these promising findings, it is still an open question whether the PSYCHOPATHY.COMP is capable of promoting emotion regulation assessed with both self-report and psychophysiological measures, which is a crucial approach to robustly assess whether the subjective perception of youth is attuned with their autonomic functioning (13–15).

### 1.1. The tripartite model of affect regulation

According to evolutionary models, humans have a set of innate motivations that allow us to survive/thrive, as well as to create and nurture affiliative bonds with others (16, 17). Grounding these motivations, research has been proposing three distinct emotion regulation systems: the threat system (quick detection of threats and automatic selection of defense responses/strategies), the drive system (activation of mobilizing positive feelings that encourage/motivate individuals to seek out for resources and pleasure), and the soothing system [allowing humans to experience states of calmness, tranquility and safeness as well as affiliation with others; (8, 16–18)]. The tripartite model of affect regulation also serves as a theoretical foundation for Compassion Focused Therapy (CFT), an evolution-based approach that acknowledges the influence of genetic, epigenetic, neural, and environmental factors in the conceptualization and treatment of mental health problems (16). According to CFT, mental health symptoms/disorders can emerge through an unbalanced functioning (e.g., under-developed/toned-down soothing system; hyper activation of the threat and/or drive systems) of these systems (16, 19). Thus, individuals face a greater risk of emotion dysregulation (16), a transdiagnostic feature in mental health problems (20).

### 1.2. Emotion (dys)regulation in young offenders

The acquisition and capacity to display adaptive emotion regulation strategies are key socio-emotional skills that develop substantially throughout adolescence (20, 21). Individuals in this developmental stage display increased frequency of risk behaviors (22, 23), greater affective fluctuations, increased negative mood (24), and increased emotional lability, a hallmark for psychopathology development (25). Thus, they are at greater risk for both internalizing and externalizing disorders (21), including conduct (CD) and/or oppositional defiant disorders [ODD; (26)]. In the externalizing spectrum, research has found that young offenders with both CD and high levels of psychopathic traits display the most severe patterns of antisocial behavior (10, 11).

Young offenders frequently develop in hostile environments (27, 28), present higher levels of negative emotions, report higher frequency of traumatic experiences (26), and most have experienced at least one adverse childhood experience (ACE; potentially traumatic events that occur during childhood and/or adolescence), with impact on both mental health and offending outcomes (29). ACEs like abuse, neglect, and household dysfunction (30), not only seem to be related with mental health symptomatology but also with specific psychophysiological patterns and reactions to various types of stimuli (29–31).

Uncaring/unsafe and threatening life experiences foster the development of a hyper-reactive threat system and/or a toned down/suppressed soothing system (8, 15). Thus, when individuals do not learn how to regulate stress through affiliative/affectionate behaviors, the attachment system can close down (32). In fact, a meta-analysis on attachment and violent offending found that insecure attachment was strongly associated with all types of criminality, even in the absence of mental health disorders (33). Within an evolutionary perspective, coping strategies to deal with ACEs, such as emotional, cognitive, behavioral, and psychophysiological, evolved to ensure survival (34–36). For young offenders, who often were confronted with multiple ACEs, research has been proposing a different emotion regulation pattern when compared to normative peers (36), which manifests itself even at the physiological level (15). In contrast, early caring and nurturing experiences stimulate the neuropathways of the soothing system, prompting the development of individuals with increased emotion regulation capacity (37).

### 1.3. Treating young offenders through CFT

Difficulties in emotion regulation is characterized by less understanding/acceptance of emotions, less control of impulsive behaviors, difficulties to behave according to goals when experiencing negative emotions and decreased capacity to use flexible emotion regulation strategies (38). Nonetheless, CFT focuses on enhancing competencies and brain systems that play a major role in regulating threatening emotions, promoting wellbeing and prosocial behavior (18) and has been shown to increase the ability to experience and resist to negative psychological states (39). Thus, CFT increases awareness and acceptance of emotions and helps individuals to better regulate emotions while decreasing their difficulties in doing so (39). Thus, available evidence suggests that emotion regulation can be nurtured and stimulated through CFT (40). This approach has been receiving growing empirical support in the treatment of several mental disorders, including severe antisocial behavior (10, 41, 42). While aiming to promote a compassionate motivation (i.e., to be sensitive to the suffering of the self and others, allied with the wisdom, strength and commitment to prevent and/or alleviate that suffering), CFT has been shown to increase the capacity for emotion regulation as well as to have a positive impact in the stimulation and development of the soothing system (18, 40, 41). Specifically, a recent clinical trial showed that a CFT-based intervention [the PSYCHOPATHY.COMP program; (10)] was able to reduce psychopathic traits and to promote a compassionate motivation among young offenders, with medium to large effect sizes (10, 12). While promoting a compassionate motivation, CFT also aims to stimulate the soothing system through the development of a more balanced autonomic nervous system [ANS; (8)]. As stated before, the soothing system associates with feelings of safeness and affiliation as well as with prosocial behaviors (18, 43, 44). This system also associates with increased pre-frontal cortex activity and has the ability to inhibit continuous threat perception (18, 45, 46). Additionally, the soothing system’s activity has been shown to be linked with compassion (47) and has the potential to regulate the emotional/behavioral outputs of both the threat and drive systems through increased activity of the vagal parasympathetic system branch of the ANS (8).

### 1.4. Autonomic functioning and emotion regulation

Increased parasympathetic nervous system activity (PNS) is associated with increased vagally mediated heart rate variability [vmHRV; (14, 15, 46–49)]. The vagus nerve acts as a “brake” in the sympathetic nervous system (SNS). It has the capacity to rapidly inhibit cardiac activity, decreasing heart rate, while increasing HRV (49). Therefore, while reflecting the interplay between the PNS and SNS activity in the cardiac output, vmHRV has been referred as an accurate marker of the autonomic activity and flexibility (44, 50–52), as well as an index of the body-mind connection (41). Additionally, vmHRV is an index of the individuals’ ability to apply adaptive emotional responses (44, 53) when facing environmental demands (53).

When the soothing system is active, PNS activity in the cardiac output is expected and should be reflected by increases in vmHRV (14). However, the lack of a safe developmental environment, with less secure attachment experiences, has been shown to be associated with the suppression of compassionate caregiving behavior (54). Due to deficient attachment development, experiences of closeness/affiliation can be conditioned to evoke feelings of threat (15, 55). Thus, kindness and compassion can be perceived as potential threats which can lead to fight/flight or shut-down responses (15, 32). At the physiological level, when triggering the soothing system, young offenders display different patterns of PNS activity in comparison to their normative peers. While community adolescents without psychopathology maintain or increase PNS activity through the triggering of the soothing system, young offenders present decreases in vmHRV, which mirrors a threat-like response to affiliative stimuli (15). Due to past experiences and memories, some inhibitors (such as fears of compassion), can prevent the compassionate motivation to be turned-on (8, 32). Thus, compassion and kindness, as well as other cues of affiliation, can activate learned defenses of attachment disruption (15). Since the physiological activation of the soothing system seems to mirror threat responses, the down regulation of the threat/drive systems (when hyper-active), can also be compromised in young offenders (15).

Several studies found that young offenders usually show increased emotion regulation difficulties, which seem to be associated with reduced parasympathetic control (25, 56, 57). Increased HRV reactivity has been related to CD behaviors during childhood and adolescence (25, 58) and, when facing a stressor, increased HRV reactivity has also been found to be a significant predictor of reoffending rates among delinquent male adolescents (59). Although research has proposed that a disturbed (re)activity of the ANS plays a major role in delinquent behavior (59–62), most works have been conducted with stressor stimuli, leaving unexplored the physiological correlates of young offenders when facing positive affiliative scenarios (15).

Interestingly, some studies reported surprising physiological correlates in participants with attachment issues. Specifically, individuals with disorganized or unresolved attachment seem to present specific physiological patterns of momentary breakdown in emotion regulation, when confronted with traumatic experiences related to attachment (63, 64). Zingaretti et al. (64) found that whereas individuals with organized attachment displayed decreases in vmHRV when confronted with negative caregiver-child attachment-related experiences, the disorganized group showed a sustained increase in vmHRV when presented with the same scenarios. It seems that individuals with disorganized attachment actively regulate their emotions during these experiences (64), using strategies such as reappraisal or suppression, which are related with increases in vmHRV (50, 64, 65). Thus, it seems that individuals with disorganized attachment had to put effort into regulating their emotions during these experiences, reflecting active attempts to suppress negative memories and regulate emotions, which requires effortful recruitment of cognitive resources [associated with increased vmHRV; (64, 65)].

### 1.5. Aims

Although different research has pointed for a disturbed ANS functioning in young offenders, less is known about their HRV patterns in potentially soothing scenarios (15). Additionally, no research so far has explored if after the delivery of a CFT intervention, the physiological correlates of the soothing system would improve. Thus, the present research aimed to test if the PSYCHOPATHY.COMP program (9) could improve emotion regulation in young offenders following a clinical trial design in Portuguese Juvenile Detention Facilities. Relevant physiological (vagally mediated HRV) and self-reported data (i.e., difficulties in emotion regulation) were collected from both treatment and control groups (in the pre-treatment, post-treatment, and 6-month follow-up periods), when the soothing system was triggered through a validated standardized procedure (14).

### 1.6. Hypotheses

H1: Based on previous research (15), both groups were expected to present decreased vmHRV when facing the soothing stimuli in the pre-treatment period (mirroring a threat-like response to this stimuli) and no differences between groups were expected regarding the physiological and the self-reported measures.

H2: For the treatment group (at post-treatment), given that the soothing scenario was expected to be processed as a threat cue and that the CFT intervention was expected to decrease difficulties in emotion regulation in these participants, vmHRV indexes were expected to be similar to the ones presented in the pre-treatment period (15). This physiological pattern was expected to be accompanied by decreases in self-reported difficulties in emotion regulation.

H3: For the post-treatment period, since participants were presented with the same soothing stimuli (as in the pre-treatment period) referring to an attachment experience, it is possible that the control group would present a dysfunctional physiological pattern (with increases in vmHRV when facing the stimuli), pointing for the recruitment of cognitive strategies associated with effortful control of negative emotional experiences (64). If so, this pattern would associate with self-reported increases in difficulties in emotion regulation.

H4: At the follow-up moment, for the treatment group, both the physiological and self-reported correlates of the soothing system triggering were expected to be maintained or improved when compared to the pre and post-treatment periods.

H5: The control group (at the follow-up moment) was expected to maintain or even worsen both the physiological and self-reported patterns when compared to both the pre and post-treatment periods.

## 2. Materials and methods

The clinical trial was conducted and designed in accordance with the Transparent Reporting of Evaluations with Non-randomized Designs [TREND Statement; (66)] and registered as a controlled trial at ClinicalTrials.gov (ID: NCT03971682).

### 2.1. Trial design and participants

The present research was conducted within the project “The Efficacy of a Compassion Focused Therapy-based Intervention in Detained Youth: A Clinical Trial,” registered in ClinicalTrials.gov (ID: NCT03971682) and a power analysis was conducted prior to data collection [GPower v3.1 software; (67)] showing that a total sample size of 100 detained youth was required to detect medium effects with a significance level of 0.05 and a power of 0.90 (10). For the physiological data collected for the present research, a power analysis was also performed, showing that a total sample size of 94 participants was required to detect medium effect sizes with a significance level of 0.05 and a power of 0.80. Since the present research was nested in the above-mentioned project, physiological data collection was conducted with the eligible participants. Thus, participants were selected from all six Portuguese juvenile detention facilities and were required to be aged between 14 and 18 years old. For both treatment and control groups, the following exclusion criteria were considered: (1) non-Portuguese speaking; (2) less than 12 months of detention counting from the beginning of the program (considering both the length and assessment period of the PSYCHOPATHY.COMP); (3) presence of cognitive impairment; (4) presence of psychotic symptomatology [given that the experiential exercises performed in the program were contraindicated for psychotic patients; (10)]; (5) presence of autism spectrum disorders (the program was not designed considering the social impairments of these youth); (6) obesity [body mass index (BMI) > 30 kg/m^2^; given not only the reduced parasympathetic activity to the heart but also that obese individuals display disruptions to the normal maturation of the cardiac autonomic control; (68)]; (7) presence of cardiovascular diseases given its interference in cardiovascular function that could introduce noise/artifacts to the data being collected; (8) uncorrected visual/hearing impairments (e.g., lack of glasses or hearing aid), in order to assure the participants’ similar execution of the standardized procedure without the interference of uncontrolled variables.

Given that research has pointed that the association between Conduct Disorder (CD) and psychopathic traits is a predictor of worse prognosis (1, 11), the presence of CD as the main diagnosis was considered as inclusion criteria for this study. Although the sex of the individual seems to play a relevant role in the intensity of emotional responses (69), in the preferred emotion regulation strategies (70), as well as in the autonomic patterns displayed by individuals (71), female detained youth were excluded from this research as they account for a small percentage of detained adolescents in Portuguese juvenile detention facilities (26), which would make any comparative analyses between samples an understatement and difficult to interpret. Although it was asked for, detailed information regarding medication intake was not provided to the research team as this information is considered confidential.

### 2.2. Interventions

The PSYCHOPATHY.COMP program (9), is a CFT-based intervention delivered in an individual format. Through the development of a compassionate motivation, it was specifically designed to reduce psychopathic traits and antisocial behavior in young offenders [see (72) for an in-depth description]. Although similar to other CFT programs regarding the strategies of change as well as the inclusion of compassionate mind training [CMT; (8)], this intervention was specifically tailored to attend to the specificities of detained youth (10). Additionally, since detained youth often present poor treatment engagement, this program highlights motivational interviewing strategies (73).

The PSYCHOPATHY.COMP delivers 20 manualized sessions (one session per week with a 60-min duration). The program itself is organized into four successive modules following a progressive strategy of change: (1) the basics of our mind; (2) our mind according to CFT; (3) Compassionate Mind Training; and (4) recovery, relapse prevention, and finalization (10). Module 1 offers insights regarding the evolution-based nature of human emotions, motives and needs as well as instinctive responses to threats (16). In this module, detained youth are encouraged to understand that although we cannot change events, we are able to change the way we relate/act on them. CMT is introduced at this stage as a way to build the participants’ compassionate mind and awareness (72, 74).

While continuing CMT, Module 2 is focused on the functioning of the human mind and body according to the theoretical framework of CFT (9, 16). Detained youth are compassionately guided to discover that we can make conscious actions with increased awareness about our functioning and that we are “just one version of ourselves.” Thus, the notion of determinism associated with evolution, genetics, or contextual experiences are demystified. Within this module, youth are introduced to the affect regulation systems (i.e., threat/drive/soothing) and their emotional, cognitive, and behavioral outputs, as well as to shame and safety strategies (16, 36). In Module 3, the intervention is explicitly focused on CMT. Participants are enrolled in a set of experiential exercises aiming to expose them to the triggering of the threat system, including exposure to anger and to compassion [as these youth usually display greater fears of compassion: fears of receiving compassion from others; fears to be compassionate toward others; and fears of being compassionate to the self; (12)]. Thus, detained youth are allowed to gradually experience and understand the outputs of the threat system while searching for compassionate strategies to bear and manage their own distress in adaptive ways (9). Finally, Module 4 aims to prevent relapse through the revisiting of compassionate motivations (9). Detained youth are encouraged to accept that suffering is a life constant and that throughout the intervention process, they were offered compassionate emotion regulation strategies that they can use to cope with suffering (9, 74).

In the present research, the treatment group attended the PSYCHOPATHY.COMP program for about 6 months and controls received the treatment as usual (TAU) delivered in Portuguese Juvenile Detention Facilities (which encompasses around 20 individual counseling sessions). The treatment group did not attend TAU and controls did not attend the PSYCHOPATHY.COMP program.

### 2.3. Measures

Mini-International Neuropsychiatric Interview for Children and Adolescents [MINI-KID; (75); Portuguese Authorized Version by Rijo et al. (26)]. The MINI-KID is a structured clinical interview that assesses the DSM-5 (76) diagnoses, both in children and adolescents. The MINI-KID also considers the impairment and frequency of the symptoms. It is as a short and accurate measurement tool to diagnose mood disorders, anxiety disorders, substance-related disorders, tic disorders, disruptive disorders, attention-deficit hyperactivity disorder, psychotic disorders, eating disorders, and adjustment disorders. The interview also accounts for a section specific for the screening of autism spectrum disorders. All questions in this instrument are presented in a binary (i.e., yes/no) format. The MINI-KID also includes items to address medical, organic, and/or drug causes for disorders. It allows the interviewer to decide which disorder should be consider as the main diagnosis. The interview takes between 30–90 min on average to administer and inter-rater reliability was found to be excellent for all mental health disorders assessed, except for dysthymia (75).

International Affective Pictures System [IAPS; (77); Portuguese version by Soares et al. (78)]. The IAPS is an international database of affective stimuli used in emotional/attentional processes research. Stimuli are scored using three criteria (77): (1) the emotional valence of the stimuli (positive or negative); (2) the perception of control of the participants to the stimuli; (3) the degree of arousal that the stimuli triggers in the participant. Based on these criteria (78), 86 emotionally neutral pictures were selected for the resting and recovery phases (for each phase, 60 randomly selected images were presented) of the standardized procedure used in this research (see Section “Procedures”). The average valence and arousal of the selected visual stimuli were 5.07 and 3.95, respectively, agreeing with the assessment criteria for emotionally neutral pictures (14, 15, 78).

### 2.4. Outcome measures–physiological and self-reported measures of emotion regulation

#### 2.4.1. Physiological measures

The ECG signal was digitized at 2,000 Hz and inspected offline using the Kubios software, (79, 80). Successive R waves identified by an automatic beat detection algorithm were visually inspected and a threshold-based artifact correction algorithm (very low: 0.45 s) was applied. Both time and frequency domain indexes of HRV were obtained for each period of interest of the standardized procedure (SP–i.e., resting, reactivity, and recovery phases). For time domain, the RMSSD (square root of the mean of the sum of the squares of differences between adjacent NN intervals) was computed (51, 81). For frequency domain, the absolute power of the high-frequency band (0.15–0.4 Hz; HFms^2^) was computed.

**FIGURE 1:**
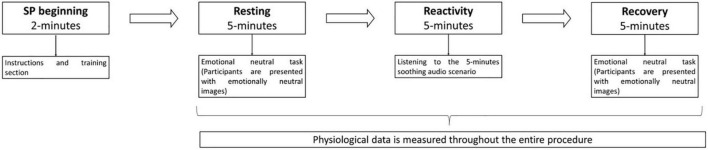
Schematic presentation of the standardized procedure.

For the physiological assessment, a standardized procedure (SP), validated for the Portuguese adolescent population [for a detailed description, see (14)], was presented to all eligible participants. The SP was based on the triple R paradigm (82) and thus composed of a resting, reactivity, and recovery phase for the triggering of the soothing system. The SP begins with an instruction section that provides participants with all relevant information for its correct execution. Then, the resting phase is initiated, and participants were presented with emotionally neutral stimuli which consisted of neutral images retrieved from the International Affective Pictures System [IAPS; (77, 78); see Section “Measures”]. The resting phase had the length of exactly 5 min, following guidelines for short-term HRV assessment (81). Each presented image lasted for 5 s, and they were randomly presented to participants (the resting phase was composed of 60 images). Next, participants performed the reactivity phase, where a 5-min audio scenario aiming to trigger the soothing system (14) was presented. The scenario was developed considering the life stage of participants and represented a situation in which an adolescent is holding a family baby that has fallen sleep. The environment is depicted as a calm, warm, and soothing one. At some point, the baby awakes but with some gentle, kind, and warm words, the baby falls sleep again, in the arms of the adolescent. Immediately after reactivity, the 5-min recovery phase begins. Similar to the resting phase, participants were presented with emotionally neutral stimuli (see Figure 1). Thus, a continuous measurement of the physiological data across the SP was assured. The described SP lasted exactly 17 min.

Regarding the collected physiological measures across the SP, analyses of normality were performed, and following the guidelines of Laborde et al. (81), when data was not normally distributed, natural logarithmic transformations were performed [Shapiro–Wilk analyses revealed non-normal distributions for these variables across all phases of the SP (see the Supplementary file for analyses in normality); both the RMSSD and the HFms^2^ were transformed with natural logarithms for all phases of the SP]. Given that both the RMSSD and the absolute power of the HF band index cardiac vagal tone, they are considered reliable markers of PNS activity (81). Increased scores in these indexes are associated with increased PNS activity.

#### 2.4.2. Self-report measure

Difficulties in Emotion Regulation Scale–Adolescent Version [DERS-AV; (38); Portuguese version for adolescents adapted from the Portuguese version for adults by Coutinho et al. (83)]. The DERS-AV is 36-item self-report measure that assesses multiple aspects of difficulties in emotion regulation. In its original version (38), this instrument is composed of six dimensions: Non-Acceptance of Emotional Responses (non-acceptance); Difficulties Engaging in Goal-Directed Behavior (goals); Impulse Control Difficulties (impulse); Lack of Emotional Awareness (awareness); Limited Access to Emotion Regulation Strategies (strategies); and Lack of Emotional Clarity (clarity). Participants are asked to rate in a five-point scale how often the items apply to them from 1 (almost never) to 5 (almost always). Higher scores, after converting all reverse-coded items, both for the subscales and the overall score, indicate greater difficulties in ER. In the original study, internal consistency was excellent for the overall scale (α = 0.93) and good for the subscales [ranging from 0.80 for awareness, to α = 0.89 for goals; (38)]. For the Portuguese adolescents’ validation study, internal consistency was excellent for the total score (α = 0.92) and ranged from 0.73 (awareness) to 0.87 (strategies) for its six dimensions. For the present study, internal consistency for the total score was 0.89 and ranged from 0.71 (clarity) to 0.88 (non-acceptance) for its six dimensions.

### 2.5. Procedures

Prior to data collection, this work was approved by the ethics committee of the Faculty of Psychology and Educational Sciences of the University of Coimbra as well as by the Portuguese Ministry of Justice.

Portuguese Juvenile detention facilities usually have no more than 150 youths that face from 6 to 36 months of detention. Additionally, around 10 youth enter and leave the facilities each month (10). Thus, it is difficult to randomly assign participants to conditions. In an effort to minimize such an impact, to maximize time/human resources and the quality of the trial design (84), as well as to account for experimental loss or ineligible participants (meeting the power analyses results for the present research; see Section “Trial design and participants”), the research team opted to assign each group with around 60 participants. The first 60 youth entering the facilities during the research period were assigned to the treatment group and the following 60 to the control group. Thus, participants were only allocated to the control group after the treatment group was completed. After 1 month of detention (which was considered as a necessary adaption period of youths to the juvenile detention facility), a first contact between the research team and the eligible participants was carried out, aiming to explain the research goals and the PSYCHOPATHY.COMP program to the youths. No payment nor extra credit were offered, and all ethical assurances were provided to participants. Confidentiality and anonymity regarding their responses were guaranteed. Youths were also informed that the voluntary nature of their participation in the program would not impact their sentences nor their school grades. After, they were invited to participate in the study and informed if they were allocated to the treatment or control group. For participants with 18 years, both written and verbal consent was obtained. For participants younger than 18 years old, verbal informed assent was obtained in addition to the youth’s legal guardians written consent.

To those who agreed to participate, a structured clinical interview (MINI-KID) was conducted. Within the interviewing process, inclusion/exclusion criteria were also assessed, assuring the participants’ eligibility to integrate the study as well as to perform the SP. Eligible participants were then assigned to the treatment or control groups. Youths allocated to the treatment group were assessed before the first session of the program (pre-treatment assessment), after the ending of the program (post-treatment assessment), and 6 months after treatment completion (6-months follow-up assessment). Participants allocated to the control group were assessed in the same time intervals. In each assessment point, all participants were assessed through self-report (i.e., DERS-AV; see Section “Measures”) and physiological measures of emotion regulation.

Prior to the execution of the SP, participants were asked to restrain themselves, for at least 2 h, from extreme physical activity and from the consumption of caffeine and other substances that might influence autonomic activity. To reduce environmental interferences, the SP was always performed in the presence of a research team member in rooms with reduced noise sources.

For the psychophysiological data measurement, the *Firstbeat Bodyguard 2* device was employed. This non-invasive device allows for real time HRV measurement through two disposable electrodes. One attached to the right side of the body, bellow the collar bone, and the other attached to the left side of the rib cage. Participants were asked to sit in front of the computer with their knees at a 90° angle, with both feet on the floor and hands on their thighs (81). During the SP execution, soundproof headphones were hooked up to participants, aiming to reduce external noise stimuli. Additionally, all participants were instructed to remain as still as possible during the SP execution, reducing potentially cardiac activity artifacts in the recorded data. The ECG was continuously recorded throughout the SP.

### 2.6. Data analysis

For the present study, preliminary analyses were conducted comparing the treatment and control groups on relevant variables including demographics, and in the outcome self-reported and physiological measures. Depending on the type of data, both independent-samples *t*-tests or chi-square tests were performed. Additionally, based on the interquartile range rule provided by the SPSS software, preliminary analyses were conducted to identify outliers within the physiological data. All data analyses were conducted with the IBM SPSS Statistics v24.0 Software and given the longitudinal design of this research, the intervention effects were tested according to the intention to treat paradigm.

To investigate for treatment effects of the PSYCHOPATHY.COMP across time (pre-treatment, post-treatment, and follow-up) in the physiological correlates of the SP, a three-factor [i.e., Condition (Treatment vs. Control); Assessment (i.e., pre-treatment, post-treatment, and follow-up); and SP phases (i.e., resting, reactivity, and recovery phases)] mixed multivariate analyses of variance (MANOVA) was performed (this analysis was conducted in order to explore hypotheses 1–5 in what regards the physiological patterns of participants). For the self-reported data, also to investigate treatment effects, two-factor [i.e., between subjects—Condition (Treatment vs. Control)—and within subjects—Assessment (i.e., pre-treatment, post-treatment, and follow-up)] analyses of variance (ANOVA) were performed (these analyses were conducted in order to explore hypotheses 1–5 in what regards the self-reported patterns of participants).

Given the different nature of the constructs under assessment, separate analyses were performed, one with the physiological measures and the other with the self-reported measure as dependent variables. Effect sizes were computed using partial eta square (*η*_p_^2^), with *η*_p_^2^ = 0.01 referring to a small effect size, 0.06 to a medium effect size, and 0.14 to a large effect size (85). For the exploration of baseline differences between groups, all *post hoc* testing was conducted using Bonferroni–Holm’s adjustment for multiple comparisons.

## 3. Results

### 3.1. Recruitment and retention

**FIGURE 2:**
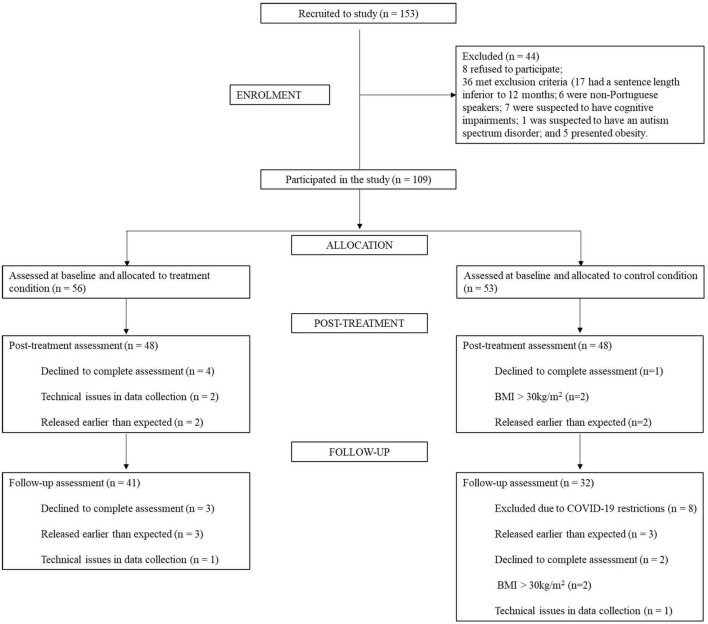
Flowchart of detained youth participation.

Initially, a total of 153 detained youths were contacted and invited to participate in the study (see Figure 2). Following the exclusion criteria assessment, through the consulting of the youth juvenile justice records and/or through the clinical interviewing process, 44 (28.8%) participants were excluded: 8 (5.2%) refused to participate and 36 (23.5%) met exclusion criteria: 17 (11.1%) had a sentence length inferior to 12 months; 6 (3.9%) were non-Portuguese speakers; 7 (4.6%) were suspected to have cognitive impairments; 1 (0.7%) was suspected to have an autism spectrum disorder; and 5 (3.3%) presented obesity. Therefore, for the initial selection, 109 (71.2%) detained youth completed the assessment at the pre-treatment period (baseline assessment) and were allocated to the treatment or control group.

Initially, 56 participants were allocated to the treatment group (Facility 1: 7 youths; Facility 2: 6 youths; Facility 3: 9 youths; Facility 4: 16 youths; Facility 5: 12 youths; and Facility 6: 6 youths). For the post-treatment assessment, 48 (85.7%) participants completed the PSYCHOPATHY.COMP. For the 6-months follow-up, 41 (73.2%) participants were assessed. From the initial 53 participants allocated to the control group (Facility 1: 10 youths; Facility 2: 9 youths; Facility 3: 6 youths; Facility 4: 17 youths; Facility 5: 5 youths; and Facility 6: 6 youths), 48 (90.6%) completed the post-treatment assessment and 32 (60.4%) also completed the follow-up assessment (see Figure 2 for detailed information regarding dropouts). It should be stated that due to the COVID-19 restrictions and government mandatory curfew, researchers were not allowed to enter the juvenile detention facilities for some time which translated in significant losses within the clinical trial for the control group (for the follow-up assessment; see Figure 2).

### 3.2. Baseline differences

To explore differences between the treatment and control groups that might confound the interpretation of results in the main analyses, a set of preliminary analyses were conducted to ascertain for baseline similarity. Most of the sample did not report visual problems (98.2%) and 1.8% had their eyesight problems corrected (e.g., glasses). No participant reported hearing problems.

The mean age of the total sample was 15.88 (*SD* = 1.18) and no differences [*t* (107) = 0.536, *p* = 0.593] between the treatment (*M* = 15.82; SD = 1.15) and control groups (*M* = 15.94; *SD* = 1.23) were found. The total sample had completed on average 5.82 (*SD* = 1.26) years of education and no differences were found [*t* (107) = 0.553, *p* = 0.582] between the treatment (*M* = 5.89; *SD* = 1.08) and control (*M* = 5.75; *SD* = 1.45) groups. Regarding socio economic status (SES^1^), no differences were found between samples [χ^2^ (2) = 3.01; *p* = 0.222]. For the treatment group 94.6% of the participants belonged to a low, 5.4% to a medium and no participant had a high socioeconomic status. For the control group, 94.3% belonged to a low, 1.9% to a medium and 3.8% to a high socioeconomic status. For BMI, the total sample presented on average 22.34 (*SD* = 2.75) and no differences [*t* (107) = 1.04, *p* = 0.300] were found between groups (treatment: *M* = 22.07; *SD* = 2.61; control: *M* = 22.62; *SD* = 2.89).

As regards the physiological measures assessed at baseline (pre-treatment), for the RMSSD index, the total sample presented an average score of 1.68 (*SD* = 0.24), and no differences [*t* (107) = 0.986, *p* = 0.326] were found between groups (treatment: *M* = 1.66; *SD* = 0.24; control: *M* = 1.70; *SD* = 0.23). Similarly, for the HFms^2^ index, the total sample presented an average score of 2.92 (*SD* = 0.47), and no differences (*t* (107) = 0.744, *p* = 0.459) were found between groups (treatment: *M* = 2.89; *SD* = 0.48; control: *M* = 2.95; *SD* = 0.47). Additionally, in order to explore if the Detention facilities from which youths were recruited had influence in their physiological correlates at baseline, one-way ANOVAs were performed for each physiological index and no statistically significant differences were found (RMSSD: *F* = 0.154, *p* = 0.978, *η*_*p*_^2^ = 0.007; HFms^2^: *F* = 0.095, *p* = 0.993, *η*_*p*_^2^ = 0.005).

Finally, for the self-reported data concerning the Difficulties in Emotion Regulation Scale (DERS-AV), no differences were found between groups at baseline (treatment: *M* = 90.28; *SD* = 22.30; control: *M* = 85.60; *SD* = 18.78) for the scale’s total score [*t* (107) = 1.136, *p* = 0.259] nor for its subscales.

### 3.3. Intervention effects in the physiological correlates of emotion regulation

**FIGURE 3:**
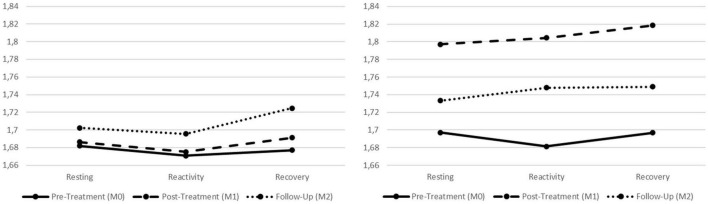
Graphic presentation of the treatment and control groups’ physiological correlates for the different phases of the standardized procedure (SP), across the pre-treatment, post-treatment, and follow-up assessment periods. Left graph refers to the treatment group and right graph refers to the control group. Both graphs refer to the square root of the mean of the sum of the squares of differences between adjacent NN intervals (RMSSD) index.

**FIGURE 4:**
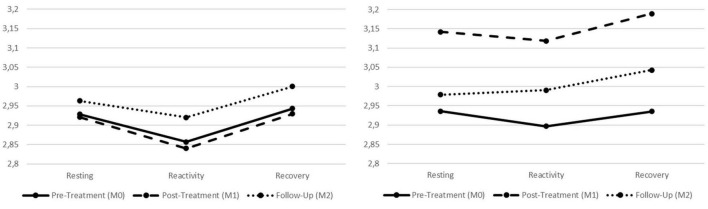
Graphic presentation of the treatment and control groups’ physiological correlates for the different phases of the standardized procedure (SP), across the pre-treatment, post-treatment, and follow-up assessment periods. Left graph refers to the treatment group and right graph refers to the control group. Both graphs refer to the HFms^2^ index.

As regards the physiological responses to the soothing scenario, initial analyses were performed considering all factors (i.e., Condition; Assessment; and the SP phases; see Section “Data analyses”) as well as the two indexes of vmHRV (i.e., RMSSD and HFms^2^). These analyses were performed in order to explore hypotheses 1–5 regarding the physiological correlates of participants. In Table 1, all physiological scores of the final sample are presented. Additionally, in Figures 3, 4, a graphical representation of the physiological data for both the treatment and control groups is also presented. No effects were found in the Condition X Assessment X SP phases interaction (Wilks’ λ = 0.973, *F* = 0.961, *p* = 0.466, *η*_*p*_^2^ = 0.013; *η*_*p*_^2^ 90% CI [0.000, 0.018]). Similarly, for the Assessment X SP phases interaction, no statistically significant effects were found (Wilks’ λ = 0.983, *F* = 0.622, *p* = 0.760, *η*_*p*_^2^ = 0.009; *η*_*p*_^2^ 90% CI [0.000, 0.009]). However, for the Assessment X Condition interaction, statistically significant effects were found (Wilks’ λ = 0.929, *F* = 2.635, *p* = 0.034, *η*_*p*_^2^ = 0.036; *η*_*p*_^2^ 90% CI [0.001, 0.066]) with a small effect size. Within the Assessment X Condition interaction, univariate analyses also revealed statistically significant effects for both vmHRV indexes: a small effect size for the RMSSD (*F* = 3.325, *p* = 0.039, *η*_*p*_^2^ = 0.045; *η*_*p*_^2^ 90% CI [0.002, 0.103]) and a medium effect size for the HFms^2^ (*F* = 4.574, *p* = 0.012, *η*_*p*_^2^ = 0.061; *η*_*p*_^2^ 90% CI [0.008, 0.125]).

**TABLE 1:** Mean scores and standard deviations of the physiological correlates for the treatment and control groups at resting, reactivity, and recovery across the assessment periods.

	Control group			Treatment group		
	Resting	Reactivity	Recovery	Resting	Reactivity	Recovery
	***M* (SD)**	***M* (SD)**	***M* (SD)**	***M* (SD)**	***M* (SD)**	***M* (SD)**
**Pre-treatment**						
RMSSD	1.70 (0.26)	1.68 (0.29)	1.70 (0.26)	1.68 (0.23)	1.67 (0.25)	1.68 (0.25)
HFms^2^	2.94 (0.54)	2.90 (0.55)	2.94 (0.52)	2.93 (0.48)	2.86 (0.53)	2.94 (0.47)
**Post-treatment**						
RMSSD	1.80 (0.20)	1.81 (0.21)	1.82 (0.20)	1.69 (0.27)	1.67 (0.26)	1.69 (0.27)
HFms^2^	3.14 (0.39)	3.12 (0.44)	3.19 (0.41)	2.92 (0.58)	2.84 (0.54)	2.93 (0.57)
**Follow-up**						
RMSSD	1.73 (0.22)	1.75 (0.24)	1.75 (0.22)	1.70 (0.23)	1.70 (0.25)	1.72 (0.25)
HFms^2^	2.98 (0.44)	2.99 (0.48)	3.04 (0.49)	2.96 (0.50)	2.92 (0.52)	3.00 (0.52)

M, mean; SD, standard deviation; RMSSD, square root of the mean of the sum of the squares of differences between adjacent NN intervals; HFms^2^, absolute power of the high-frequency band. Logarithmic transformations were performed in RMSSD and HFms^2^ indexes.

Given that statistically significant results were found in the Assessment X Condition interaction for both physiological indexes, there was the need to ascertain in which phase of the SP those differences were located. Since the Assessment X Condition interaction did not account for the resting, reactivity, or recovery phases of the SP, further analyses were performed for each physiological index to find if these differences were located in the resting, reactivity, or recovery phases of the SP, depending on the assessment period (i.e., pre-treatment, post-treatment, and follow-up). These analyses allowed to explore hypotheses 2–5 in more detail, considering the physiological correlates of both groups.

Therefore, further univariate analyses were conducted considering each vmHRV index individually, for the different assessment periods and depending on the participants’ condition. First, regarding the resting phase of the SP, no statistically significant interaction was found depending on the participants’ condition for the HFms^2^ (Wilks’ λ = 0.930, *F* = 2.647, *p* = 0.078, *η*_*p*_^2^ = 0.070; *η*_*p*_^2^ 90% CI [0.000, 0.165]) nor for the RMSSD (Wilks’ λ = 0.948, *F* = 1.935, *p* = 0.152, *η*_*p*_^2^ = 0.052; *η*_*p*_^2^ 90% CI [0.000, 0.139]). Participants, independent of condition, seem to maintain the same physiological correlates in the resting phase across the three assessment points. For the reactivity phase of the procedure, statistically significant effects were found for the HFms^2^ (Wilks’ λ = 0.911, *F* = 3.434, *p* = 0.038, *η*_*p*_^2^ = 0.089; *η*_*p*_^2^ 90% CI [0.003, 0.191]; medium effect size) but not for the RMSSD (Wilks’ λ = 0.923, *F* = 2.915, *p* = 0.060, *η*_*p*_^2^ = 0.077; *η*_*p*_^2^ 90% CI [0.000, 0.174]). For the recovery phase of the SP, statistically significant effects were found for the HFms^2^ (Wilks’ λ = 0.891, *F* = 4.298, *p* = 0.017, *η*_*p*_^2^ = 0.109; *η*_*p*_^2^ 90% CI [0.011, 0.216]; medium effect size) as well as for RMSSD (Wilks’ λ = 0.910, *F* = 3.483, *p* = 0.036, *η*_*p*_^2^ = 0.090; *η*_*p*_^2^ 90% CI [0.003, 0.192]; medium effect size).

In order to identify where these statistically significant differences were located, univariate analyses within each condition (i.e., treatment and control groups) were performed for each vmHRV index. For the reactivity phase of the procedure (i.e., Hypotheses 2 and 3, regarding the physiological data), the control group presented statistically significant effects for both the HFms^2^ (*F* = 4.905, *p* = 0.011, *η*_*p*_^2^ = 0.137; *η*_*p*_^2^ 90% CI [0.019, 0.254]; medium effect size) and the RMSSD (*F* = 5.676, *p* = 0.005, *η*_*p*_^2^ = 0.155; *η*_*p*_^2^ 90% CI [0.029, 0.274]; large effect size) indexes. Further *post hoc* testing for the control group revealed that both physiological indexes significantly increased from the reactivity phase of the pre-treatment to the reactivity phase of the post-treatment (HFms^2^: *p* = 0.009; RMSSD: *p* = 0.005). For the treatment group, no statistically significant effects were found when comparing the reactivity phases of the assessment moments (HFms^2^: *F* = 0.918, *p* = 0.404, *η*_*p*_^2^ = 0.022; *η*_*p*_^2^ 90% CI [0.000, 0.083]; RMSSD: *F* = 0.370, *p* = 0.680, *η*_*p*_^2^ = 0.009; *η*_*p*_^2^ 90% CI [0.000, 0.051]).

Finally, for the recovery phase of the SP (i.e., Hypotheses 4 and 5, regarding the physiological data), the control group presented statistically significant effects for both the HFms^2^ (*F* = 6.065, *p* = 0.004, *η*_*p*_^2^ = 0.164; *η*_*p*_^2^ 90% CI [0.034, 0.283]; large effect size) and the RMSSD (*F* = 7.634, *p* = 0.001, *η*_*p*_^2^ = 0.198; *η*_*p*_^2^ 90% CI [0.056, 0.320]; large effect size) indexes. Further *post hoc* testing for the control group revealed that both physiological indexes significantly increased from the recovery phase of the pre-treatment to the recovery phase of the post-treatment (HFms^2^: *p* = 0.001; RMSSD: *p* = 0.001). For the treatment group, no statistically significant effects were found when comparing the recovery phases of the assessment moments (HFms^2^: *F* = 0.731, *p* = 0.479, *η*_*p*_^2^ = 0.018; *η*_*p*_^2^ 90% CI [0.000, 0.073]; RMSSD: *F* = 1.334, *p* = 0.269, *η*_*p*_^2^ = 0.032; *η*_*p*_^2^ 90% CI [0.000, 0.101]).

In order to provide readers with graphical presentations of the physiological data across the pre-treatment, post-treatment, and follow-up assessment periods, depending on the group, a set of graphs are also provided in the Supplementary file of the present research (see Supplementary file).

### 3.4. Intervention effects in the self-reported correlates of emotion regulation

**FIGURE 5:**
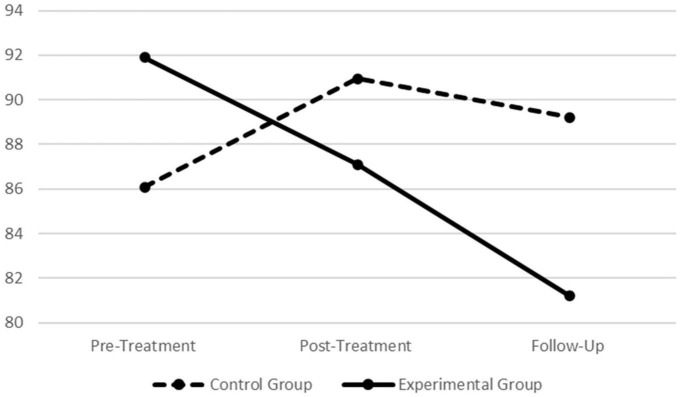
Graphic presentation of group differences in the Difficulties in Emotion Regulation Scale–Adolescent Version (DERS-AV) total score across the pre-treatment, post-treatment, and follow-up assessment periods.

**FIGURE 6:**
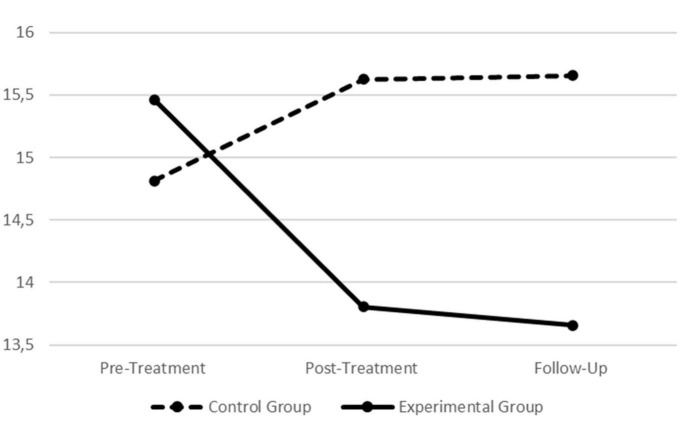
Graphic presentation of group differences in the Difficulties in Emotion Regulation Scale–Adolescent Version (DERS-AV) goals subscale across the pre- treatment, post-treatment, and follow-up assessment periods.

**FIGURE 7:**
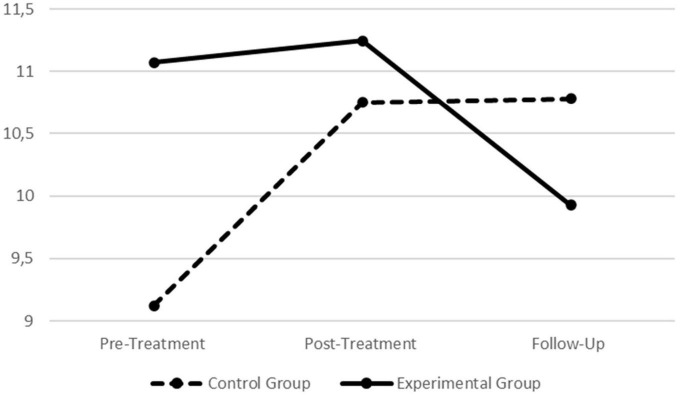
Graphic presentation of group differences in the Difficulties in Emotion Regulation Scale–Adolescent Version (DERS-AV) clarity subscale across the pre- treatment, post-treatment, and follow-up assessment periods.

In order to explore the self-report measures of difficulties in emotion regulation as well as to ascertain if the outcomes for physiological data were or were not corroborated by the self-reported measures (i.e., Hypotheses 2–5, regarding the self-reported data), a series of univariate tests were performed for the DERS-AV. All self-reported scores for the final sample are presented in Table 2. Additionally, across Figures 5–7, graphical presentations of statistically significant differences between groups for the self-reported data are also presented.

**TABLE 2:** Mean scores and standard deviations of the self-reported data for the treatment and control groups across the assessment periods.

	Control group	Treatment group
	***M* (SD)**	***M* (SD)**
**Pre-treatment**		
DERS-AV	86.09 (19.40)	91.90 (22.08)
Goals	14.81 (4.44)	15.46 (5.49)
Clarity	9.13 (3.36)	11.07 (4.05)
**Post-treatment**		
DERS-AV	90.94 (18.62)	87.10 (20.54)
Goals	15.63 (5.70)	13.80 (4.74)
Clarity	10.75 (3.59)	11.24 (4.15)
**Follow-up**		
DERS-AV	89.22 (18.10)	81.20 (23.01)
Goals	15.67 (4.77)	13.66 (5.23)
Clarity	10.78 (3.28)	9.93 (3.97)

M, mean; SD, standard deviation; DERS-AV, difficulties in emotion regulation–adolescent version.

When assessing participants scores in the different assessment periods depending on the condition (i.e., treatment vs. control), statistically significant findings were found for the scale’s total score (Wilks’ λ = 0.891, *F* = 4.299, *p* = 0.017, *η*_*p*_^2^ = 0.109; *η*_*p*_^2^ 90% CI [011, 0.216]) as well as for the goals (Wilks’ λ = 0.919, *F* = 3.075, *p* = 0.048, *η*_*p*_^2^ = 0.081; *η*_*p*_^2^ 90% CI [0.000, 0.179]) and clarity (Wilks’ λ = 0.890, *F* = 4.294, *p* = 0.017, *η*_*p*_^2^ = 0.109; *η*_*p*_^2^ 90% CI [0.011, 0.215]) dimensions (all with medium effect sizes). Thus, further univariate analyses were performed on each group separately to explore where those differences were located.

For the scale’s total score no statistically significant effects were found for the control group (*F* = 1.278, *p* = 0.286, *η*_*p*_^2^ = 0.040; *η*_*p*_^2^ 90% CI [0.000, 0.123]). For the treatment group, statistically significant effects were found (*F* = 7.111, *p* = 0.001, *η*_*p*_^2^ = 0.151; *η*_*p*_^2^ 90% CI [0.040, 0.258]; large effect size) and *post hoc* testing revealed significant decreases from the pre-treatment to follow-up (*p* = 0.003) as well as from the post-treatment to follow-up (*p* = 0.026).

For the goals dimension, no statistically significant effects were found for the control group (*F* = 0.533, *p* = 0.589, *η*_*p*_^2^ = 0.017; *η*_*p*_^2^ 90% CI [0.000, 0.077]). For the treatment group, statistically significant effects were found (*F* = 4.276, *p* = 0.018, *η*_*p*_^2^ = 0.097; *η*_*p*_^2^ 90% CI [0.010, 0.194]; medium effect size) and *post hoc* testing revealed significant decreases in this dimension from pre-treatment to follow-up (*p* = 0.048).

Finally, for the clarity dimension, statistically significant effects were found for the control group (*F* = 4.309, *p* = 0.018, *η*_*p*_^2^ = 0.122; *η*_*p*_^2^ 90% CI [0.012, 0.237]; medium effect size). *Post hoc* testing revealed that scores increased from pre-treatment to post-treatment (*p* = 0.021) and also from pre-treatment to follow-up (*p* = 0.040). For the treatment group, no statistically significant effects were found regarding this dimension (*F* = 2.203, *p* = 0.117, *η*_*p*_^2^ = 0.052; *η*_*p*_^2^ 90% CI [0.000, 0.134]).

## 4. Discussion

In recent years, research focused on the evolutionary nature of human behavior has been increasing (16, 18, 87). Constructs such as emotion regulation have been explored through these evolutionary lenses and the need to explore psychophysiological measures as useful tools to address core psychopathological mechanisms has been highlighted (13–15). Additionally, the evolutionary-based tripartite model of affect regulation (proposing the drive, threat and soothing systems), which serves as a theoretical foundation for the Compassion Focused Therapy (8), has received increasing empirical support for the conceptualization of human behavior in general (16, 18), but also for the conceptualization of emotionally dysregulated behaviors such as the ones displayed by young offenders with psychopathic traits (9, 10).

Specifically, although research has suggested different patterns of emotion regulation in severe types of psychopathologies, such as in young offenders diagnosed with conduct disorder (15, 36), no research so far has explored if a CFT intervention has the ability to improve the physiological correlates of emotion regulation in detained youth. Hence, the PSYCHOPATHY.COMP, a CFT-based intervention specifically tailored for young offenders [which was found to reduce participants’ psychopathic traits; (9, 10)], was delivered to youth placed in juvenile detention facilities. Following a clinical trial design (comparing treatment and control groups), the present research aimed to explore the physiological correlates of the soothing system in young offenders across three assessment periods (i.e., pre-treatment, post-treatment, and 6-months follow-up). To do so, detained youth, performed a standardized procedure previously validated for the Portuguese adolescent population that aimed to trigger the soothing system (14), designed following the triple R paradigm [i.e., resting, reactivity, and recovery phases; (82)]. For each assessment period, and to further strengthen this study through the comparison of both physiological and self-report data, participants also answered a measure assessing difficulties in emotion regulation.

### 4.1. Physiological correlates of the Soothing system in young offenders

When the soothing system is triggered, vagally mediated physiological responses are expected in psychologically healthy adolescents (14, 15). In the present study, vagally mediated HRV, which is an index of the ANS balance (44, 52, 53) that relates with increased capacity to display emotionally regulated behaviors (49, 53), was assessed through the RMSSD and HFms^2^ indexes. When triggering the soothing system (e.g., through affiliative stimuli), increases in these physiological indexes are expected, reflecting increases in parasympathetic activity in the cardiac output (14, 49). However, recent research (15) found that when presented with soothing stimuli, young offenders diagnosed with CD, displayed a physiological pattern different from their community peers. In fact, while the expected increases in vmHRV were found for community adolescents without psychopathology, young offenders displayed significant decreases in these indexes (15). These authors argued that affiliative experiences could be conditioned to feelings of threat and that due to past ACEs, which did not allow for an adaptive development and maturation of the soothing system, the soothing stimulus was processed as a threat cue. Thus, there was the need to explore if after a CFT-based intervention young offenders would change their perception of the soothing stimulus (processed as a threat cue) and if their physiological correlates would mirror that change.

For the present research, the main analyses did not reveal statistically significant effects when considering all factors under study. Specifically, when exploring the Condition X Assessment X SP phases, no significant interactions were found. However, statistically significant effects were found for the physiological data in the Assessment X Condition interaction. In order to ascertain in which phase of the SP the differences between treatment and control groups were located, specific analyses were conducted considering the resting, reactivity, and recovery phases individually.

First, it should be mentioned that at the pre-treatment period, for both the treatment and control groups, vmHRV (i.e., RMSSD and HFms^2^) decreased from the resting phase to the triggering of the soothing system and increased from reactivity to the recovery phase (agreeing with Hypothesis 1). This pattern is in line with previous findings (15) and stresses that for young offenders, the soothing system might be toned-down and/or underdeveloped. It seems that in the pre-treatment assessment period, both groups were not able to activate parasympathetically mediated physiological responses while being presented with an affiliative scenario. This finding also relates with Gilbert’s (8) conceptualization of fears of compassion as inhibitors that can prevent compassionate motivations to emerge. Importantly, due to past memories of attachment disruption, affiliative cues can activate learned/conditioned defenses. In fact, recent research (12) has found that not only increases in self-compassion but also decreases in fears of receiving compassion from others were the underlying mechanisms behind the changeability of psychopathic traits in young offenders, after the delivery of the PSYCHOPATHY.COMP. Additionally, for the present study, not only the physiological correlates were similar between groups, but also their self-report scores of difficulties in emotion regulation were equivalent at pre-treatment.

### 4.2. Young offenders’ physiological correlates at rest

When considering the physiological correlates of the resting phase of the SP across the assessment periods, no differences were found between groups. The control group seemed to increase their resting physiological correlates from the pre-treatment to the post-treatment, whereas the treatment group seemed to present an increasing trend in its physiological correlates from pre-treatment to follow-up. Nonetheless, these findings were not statistically significant and both groups were considered to maintain their physiological correlates for the resting phase of the procedure across the three assessment periods. At rest, decreased vmHRV has been associated with decreased capacity for emotion regulation (25, 61, 62) and meta-analytic work (88) points for an association between low HRV and worse self-control in inhibiting or diverting dysfunctional thoughts, behaviors and emotions. Additionally, lower resting HRV has been linked with hypervigilant and maladaptive cognitive responses to emotional stimuli (89).

Considering that the treatment group seemed to increase their physiological indexes across the assessment periods (from pre-treatment to follow-up), it is possible that even after the delivery of the PSYCHOPATHY.COMP program, these participants continued to improve their resting autonomic state with increases in PNS activity [i.e., vmHRV; (49, 53)]. Thus, it is possible that if vmHRV was collected in other assessment period (e.g., 12-months follow-up), statistically significant increases in the resting phases of the treatment group would be found, representing an increased capacity for emotion regulation at rest, after the delivery of the PSYCHOPATHY.COMP. On the other hand, the control group seemed to increase their resting physiological correlates from pre-treatment to post-treatment. However, from post-treatment to follow-up, this increase in resting values was apparently lost. Although interpreted with extreme caution since no statistically significant differences were found, these outcomes might reveal that at the resting phase of the post-treatment assessment, control participants were already recruiting cognitive resources [which associate with increased vmHRV; (50, 64, 65)] to deal with an expected unpleasant stimulus.

### 4.3. Triggering the soothing system-changes in young offenders’ physiological correlates

For the reactivity phases across the assessment periods, statistically significant differences were found between groups. For the treatment group (Hypothesis 2), when triggering the soothing system, their physiological correlates seemed to be maintained across the assessment periods. The PSYCHOPATHY.COMP program was designed to develop a compassionate motivation in young offenders while continuously promoting compassionate mind training (10, 9). Throughout the program, human emotions and instinctive responses to threat are demystified (11, 16, 9) and youths are encouraged to increase their awareness regarding dysregulated emotional strategies as well as their (and others) suffering, as a life constant. Additionally, with a compassionate motivation, youths are also encouraged to understand and accept that although events cannot be changed, the way we act and relate with them can be different. Therefore, given that both the present and previous research (15) have shown a potentially threatening processing of the soothing stimulus, treatment participants seem to be more aware of their suffering while accepting, but not avoiding, it. If this is the case, the interpretation of the stimulus was not changed across the assessment periods, as shown by the decreases in vmHRV when triggering the soothing system, but participants did not display the need to employ cognitive strategies to deal with it.

In contrast, for the control group (Hypothesis 3), surprising results were found concerning their physiological correlates when triggering the soothing system across the different assessment periods. For both vmHRV indexes (i.e., RMSSD and HFms^2^), scores increased from pre-treatment to post-treatment. Previous research has shown that when confronted with traumatic experiences related to attachment, individuals with attachment issues (i.e., disorganized or unresolved attachment) seem to present physiological patterns of momentary breakdown in emotion regulation (63, 64). It is possible that while the treatment group was able to better accept the threatening nature of the stimulus, the controls seemed to have recruited cognitive capacities to deal with the scenario. Indeed, strategies such as reappraisal or suppression relate with increases in vmHRV (50, 64, 65). Thus, although designed to be a soothing triggering scenario, if the stimulus is being processed as a negative attachment related experience, controls might have put effort into regulating their emotions. In order to suppress negative memories and emotions, controls seemed to recruit cognitive resources associated with increases in vmHRV (64). Given that young offenders frequently develop in hostile environments (27, 28) and are confronted with multiple ACEs which impact offending outcomes (29), it is possible that scenarios depicting affiliative behaviors might become conditioned to feelings of threat (55). Additionally, since participants were already exposed to the scenario at the pre-treatment, an anticipation of the unpleasant stimulus might have occurred at the post-treatment assessment. Additionally, although vmHRV decreased from post-treatment to follow-up, this decreasing trend was not statistically significant. It seems that controls maintained the same physiological activation at the follow-up period when compared to the post-treatment, possibly rigidly recurring to the same strategies previously employed in dealing with the unpleasant stimulus.

### 4.4. Young offenders’ physiological correlates of recovery

As regards the physiological correlates of the recovery phases across the assessment periods, no statistically significant differences were found for the treatment group (Hypothesis 4). For these participants, although there is a sustained increasing trend in vmHRV across the recovery phases, which mirrors an adaptive replenishing of vagal activity after a stressor or threat-like activation (82), this pattern was not significant. Thus, treatment participants seem to continue to process the stimulus as a threat cue while being able to physiologically recover from its activation.

However, for the controls (Hypothesis 5), a statistically significant increase in vmHRV from pre to post-treatment was found. In line with previous research, as well as with the pattern of results regarding the reactivity phases, controls seem to maintain an active recruitment of cognitive capacities to deal with the negative stimulus, even in the recovery phase (64). Indeed, when looking for the controls’ physiological pattern across the different phases of the SP (i.e., resting, reactivity, and recovery), whereas in the pre-treatment vmHRV decreases from resting to reactivity and increases from reactivity to recovery (similar to the treatment group), in the post-treatment and follow-up, an increasing pattern in vmHRV from resting to recovery was present.

### 4.5. Findings on self-reported difficulties in emotion regulation

Although findings of the present research are complex, data from the self-report measures seem to corroborate the pattern of results for the physiological data (Hypotheses 2–5). Specifically, the DERS-AV proposes a functionalistic approach to emotion regulation, stating that adaptive emotion regulation processes involve a broad repertoire of skills which include the flexibility to appropriately select emotion regulation strategies in accordance with situational demands (90, 91). Thus, higher scores in this scale, represent higher levels of difficulties in emotion regulation and relate with an inflexible and rigid selection of maladaptive emotion regulation strategies despite the demands of a specific environment (38). For the present research, differences between groups were found for the DERS-AV total score. From pre-treatment to follow-up, the treatment group presented a statistically significant decreasing trend in this scale. It seems that the PSYCHOPATHY.COMP program was able to produce change in participants, decreasing their overall difficulties in emotion regulation. However, for the control group, no differences were found, and participants seem to maintain the same level of difficulties in emotion regulation across the different assessment moments.

As regards the goals dimension of the scale, statistically significant differences were found between groups. Increased scores relate with the avoidance of internal experiences and attempts to control both the experience and expression of unpleasant emotions which would result in the non-acceptance of emotional responses, as well as difficulties in engaging in goal directed behaviors (38, 92). For the treatment group, statistically significant decreases in this subscale were found from pre-treatment to follow-up. The PSYCHOPATHY.COMP aims to increase the emotional acceptance of threatening stimuli while promoting a compassionate motivation in detained youth. Participants gradually experience and understand the outputs of the threat system while searching for compassionate strategies to manage their suffering in adaptive ways (9). Thus, the treatment group seemed to be able to better engage and accept the unpleasant emotional experience, without needing to avoid it or recruit maladaptive strategies to deal with the soothing stimulus (which is being processed as threatening). Additionally, for the control group, no differences were found for this subscale across the assessment periods. Although scores increased from pre-treatment to post-treatment and were maintained at follow-up, given the non-significance of this pattern, controls seemed to maintain their initial levels of avoidance of internal experience, as well as maladaptive attempts to control unpleasant emotions and engage in goal directed behaviors.

Finally, for the DERS-AV clarity subscale, statistically significant differences between groups were also found. This subscale relates with the lack of emotional clarity and awareness in monitoring and evaluating emotional experiences (38, 93). For the treatment group, although seemingly decreasing from the pre and post-treatment to the follow-up period, this pattern of results was not statistically significant and treatment participants seemed to maintain their initial levels of emotional clarity. However, controls significantly increased their lack of emotional clarity across the different assessment periods, which points for the PSYCHOPATHY.COMP program ability to prevent the deterioration of this relevant skill for adaptive emotion regulation in the treatment group.

As previous research has pointed out, difficulties in emotion regulation (assessed through the DERS) are negatively correlated with resting vmHRV (94, 95) and this relation not only associates with the inability to behave following personal goals (94) but also with anxiety and ruminative tendencies (95). Taken together, the physiological and self-report data are congruent and complement each other for a deeper understanding and interpretation of results, while offering support for the ability of a CFT-based program in improving emotion regulation in young offenders diagnosed with CD.

### 4.6. Overall efficacy of the PSYCHOPATHY.COMP

The PSYCHOPATHY.COMP seems to be able to improve emotion regulation in young offenders when assessed both through physiological and self-report measures. At the self-report level, treatment participants were able to decrease their overall difficulties in emotion regulation, and this change was maintained at the follow-up period. This finding translates at the physiological level with participants being able to better accept the unpleasant affiliative stimulus, without needing to employ maladaptive coping strategies. These are promising findings offering support for the efficacy of the PSYCHOPATHY.COMP program and should be considered in future clinical interventions with young offenders. At initial stages of the intervention, trying to promote and develop the soothing system might increase resistances and fears of compassion given that youths might activate past memories of disruptive attachment. Nonetheless, with gradual and guided compassionate exposure to the activation of the threat system, youths can better accept the unpleasant nature of such stimuli while adaptively recovering from them. Thus, it seems that the need to actively recruit maladaptive coping strategies in these scenarios is diminished and the nurturing/development of the soothing system is potentially facilitated throughout the intervention. Clinically, following the youth’s personal goals, cognitive restructuring of how unpleasant affiliative stimuli are perceived, and the practice of more effective/adaptive coping skills should be aimed for.

As regards the controls, both self-report and physiological data support an increase in emotion regulation difficulties across time. Importantly, physiological data seem to suggest that without the program, controls rigidly employ the same maladaptive strategies over time and were not able to effectively process or recover from the soothing system scenario in an adaptive manner. The desensitization of threat conditioning in relation to the triggering of the soothing system might be difficulted. Therefore, if triggering the soothing system is required in order to down-regulate hyper-activation of both the threat and drive systems but rigid maladaptive strategies are recurrently employed to deal with the activation of the soothing (processed as threat), the development of a more balanced autonomic system might be compromised.

### 4.7. Limitations and future research

Although this study is pioneering and innovative in nature, it is not free of limitations. First, the initial lack of randomization of participants regarding their allocation to the treatment or control groups, although justifiable, should be acknowledged. Also, due to the COVID-19 pandemic as well as the mandatory governmental restrictions, experimental loss was mostly suffered in the follow-up moment for the control group. This issue decreased the number of participants included in the analysis and it is possible that with an increased sample size, additional statistically significant findings would be found.

Regarding the used physiological measures (i.e., RMSSD and HFms^2^), although thought to be robust to respiratory influence (81), respiration was not controlled for, and this should be recognized as a limitation of the present research. Additionally, in order to explore the full range of autonomic functioning in young offenders, future research should include measures of sympathetic functioning (e.g., electrodermal activity; pre-ejection period). The effort to control not only for medication intake but also for other specificities within young offenders’ life trajectories (e.g., type of index crime and previous sentences) should be pursued and its effects on the physiological correlates explored.

Importantly, increased vmHRV has been frequently reported as an indicator of increased emotion regulation capacity. The present study stresses the need to surpass this simplistic approach to the physiological data discussion as well as the need to complement it with relevant self-report measures in order to clarify this field. Congruently, as in the present research dispositional traits were not collected (which could have influenced participants’ emotional experience throughout the standardized procedure), future research should include these measures, while exploring their influence in the physiological patterns of young offenders.

Finally, although the collected self-report data helped to better understand the physiological findings, it is now clear that future studies assessing autonomic functioning in young offenders should also include measures specifically designed to assess attachment styles and coping strategies. Researchers should explore the role of these constructs on the complex physiological patterns displayed by young offenders when their soothing system is triggered, and these considerations should be taken into account within clinical settings.

## Data availability statement

The datasets presented in this article are not readily available because the dataset is considered anonymous. Sharing of the data might be available under specific conditions following contact with the corresponding author. Requests to access the datasets should be directed to RS, rubenabrantessousa@gmail.com.

## Ethics statement

The studies involving human participants were reviewed and approved by Ethics committee of the Faculty of Psychology and Educational Sciences of the University of Coimbra; Portuguese Ministry of Justice. Written informed consent to participate in this study was provided by the participants’ legal guardian/next of kin.

## Author contributions

RS, DRS, NP, PG, and DR contributed to the conception and design of the study. RS and DRS organized the database and performed the statistical analysis. RS, DRS, and DR wrote the first draft of the manuscript. All authors contributed to the manuscript revision, read and approved the submitted version.

## Footnotes

1 Socioeconomic status (SES) was measured by parents’ profession, considering the Portuguese professions classification (86). Examples of professions in the high SES group are judges, higher education professors, or MDs; in the medium SES group are nurses, psychologists, or schoolteachers; and in the low SES group are farmers, cleaning staff, or undifferentiated workers.
