# Supplementary material for: At the heart of change: Differences in young offenders’ HRV patterns after the delivery of the PSYCHOPATHY.COMP program
Source: Front Psychiatry. 2023 Jan 10;13:1032011. doi: 10.3389/fpsyt.2022.1032011 (PMC9872126; doi:10.3389/fpsyt.2022.1032011)
Supplement: Supplementary file 1 [file Data_Sheet_1.docx]

**Supplementary File**

**Figure 1**

**
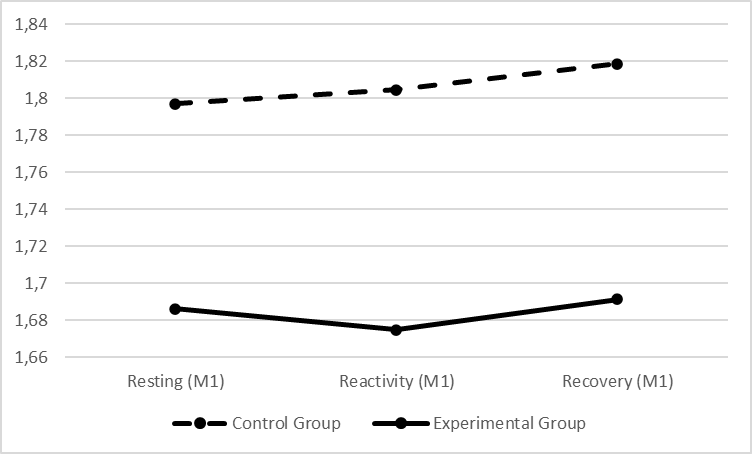
**Graphic presentation of RMSSD differences between groups across the SP phases (i.e., resting, reactivity and recovery) for each assessment period. Left graph refers to pre-treatment, mid graph refers to post-treatment and right graph refers to follow-up.

**
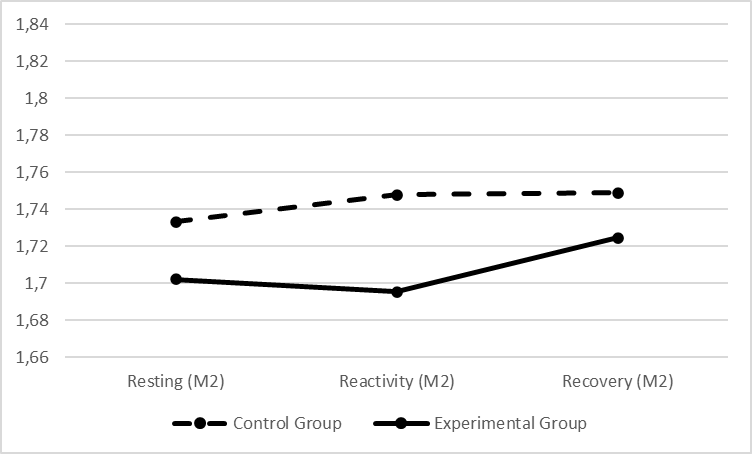

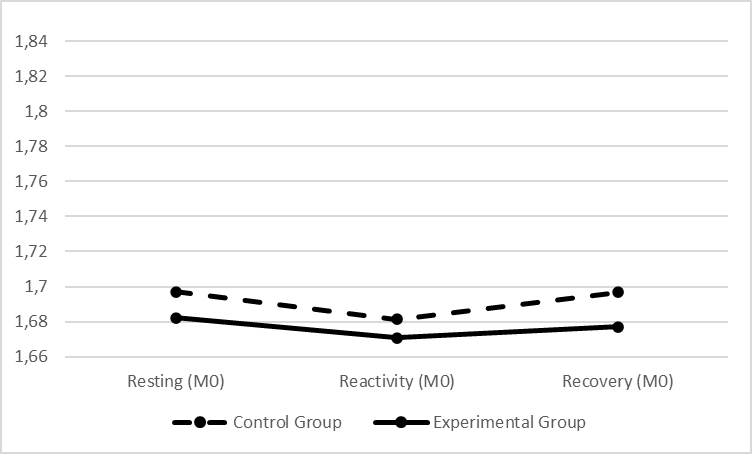
**

**Figure 2**

Graphic presentation of HFms^2^ differences between groups across the SP phases (i.e., resting, reactivity and recovery) for each assessment period. Left graph refers to pre-treatment, mid graph refers to post-treatment and right graph refers to follow-up.


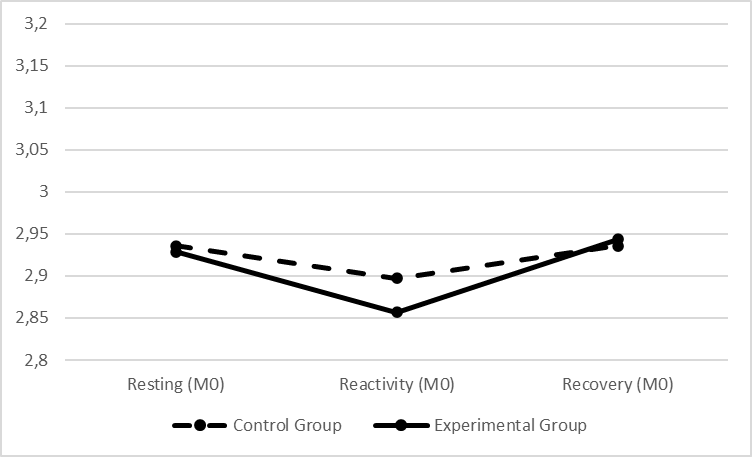

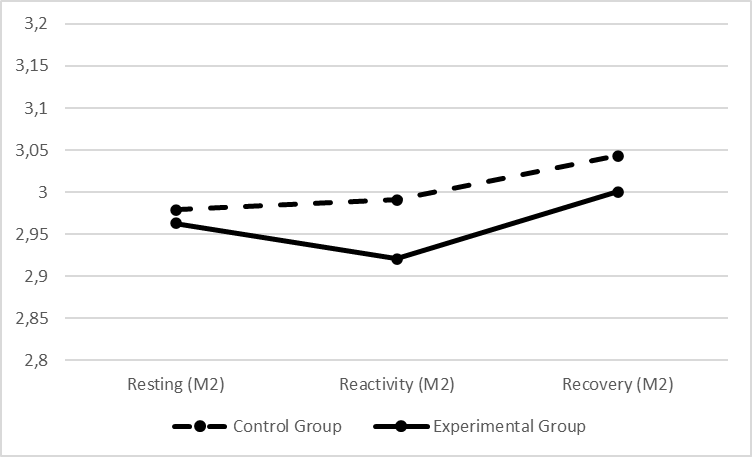

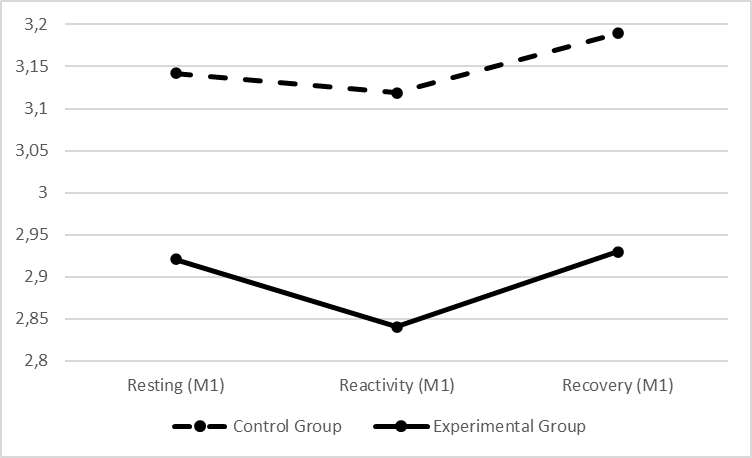


**Figure 3**


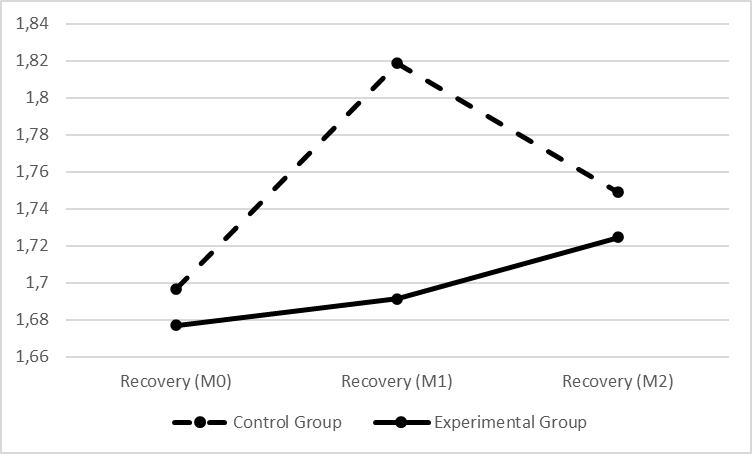

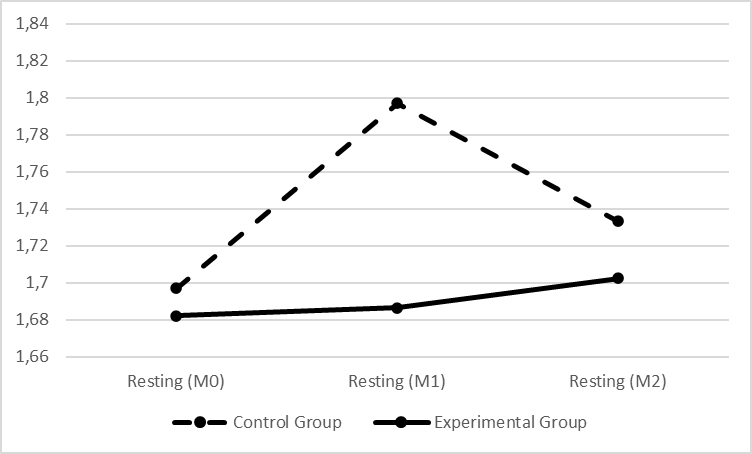
**
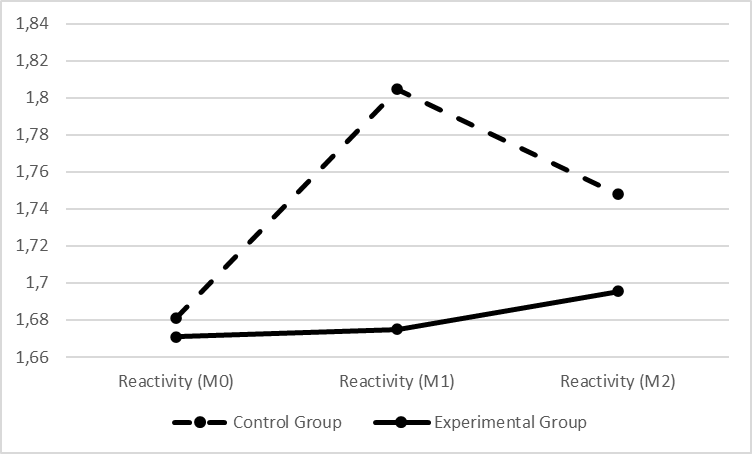
**Graphic presentation of RMSSD differences between groups across the assessment periods (i.e., pre-treatment, post-treatment and follow-up) for each phase of the SP. Left graph refers to the resting phases, mid graph refers to the reactivity phases and right graph refers to the recovery phases.

**Figure 4**


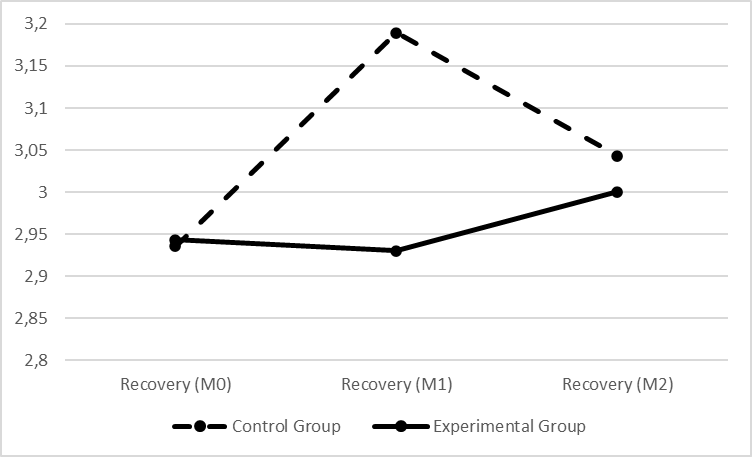

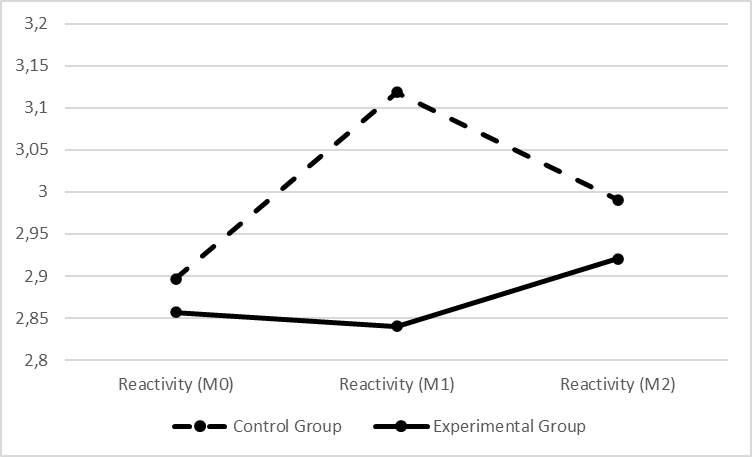

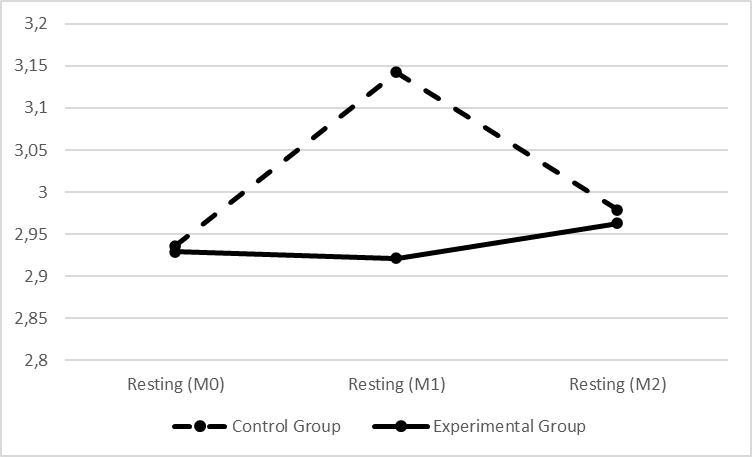
Graphic presentation of HFms^2^ differences between groups across the assessment periods (i.e., pre-treatment, post-treatment and follow-up) for each phase of the SP. Left graph refers to the resting phases, mid graph refers to the reactivity phases and right graph refers to the recovery phases.

Analyses of normality (Shapiro-Wilk test) for the physiological data across all phases of the Standardized Procedure:

**RMSSD:**

**Pre-treatment:**

Resting: W = .954 (109); p = .001

Reactivity: W = .948 (109); p < .001

Recovery: W = .941 (109); p < .001

**Post-treatment:**

Resting: W = .944 (96); p < .001

Reactivity: W = .936 (96); p < .001

Recovery: W = .937 (96); p < .001

**Follow-up:**

Resting: W = .879 (73); p < .001

Reactivity: W = .890 (73); p < .001

Recovery: W = .903 (73); p < .001

**HFms^2^:**

**Pre-treatment:**

Resting: W = .813 (109); p < .001

Reactivity: W = .797 (109); p < .001

Recovery: W = .809 (109); p < .001

**Post-treatment:**

Resting: W = .746 (96); p < .001

Reactivity: W = .797 (96); p < .001

Recovery: W = .789 (96); p < .001

**Follow-up:**

Resting: W = .650 (73); p < .001

Reactivity: W = .682 (73); p < .001

Recovery: W = .686 (73); p < .001
